# Vascular Considerations for Patients Receiving Amyloid Immunotherapy

**DOI:** 10.1093/geroni/igaf122.1009

**Published:** 2025-12-31

**Authors:** Steven Greenberg

**Affiliations:** Harvard University, Cambridge, Massachusetts, United States

## Abstract

Amyloid-related imaging abnormalities (ARIA) in the form of edema/effusions (ARIA-E) or hemorrhagic lesions (ARIA-H) are the most impactful adverse event associated with anti-amyloid immunotherapy for Alzheimer disease. Most ARIA events are mild or silent, but a small subset can be severely symptomatic or fatal. The pathophysiologic mechanisms for ARIA appear centered around interactions of β-amyloid peptide (Aβ) with the cerebrovasculature as it transits from plaques to the perivascular space or through direct antibody-Aβ interactions after deposition in arterioles as cerebral amyloid angiopathy (CAA). The key role of the vasculature in generating ARIA creates challenges for vascular clinicians, including safety of immunotherapy for individuals with CAA markers, whether immunotherapy-treated patients can also receive anticoagulants or thrombolytics, and treatment of severe ARIA. Among key vascular that have emerged from clinical trial data and adverse event reports are that 1) knowledge about ARIA should be disseminated throughout clinical teams providing stroke care, 2) ARIA risk can be stratified by pretreatment markers of CAA and incorporated into risk-benefit discussions, 3) the suggestive link between elevated blood pressure and ARIA risk supports close adherence to hypertension guidelines, 4) concomitant anticoagulation may increase risk of symptomatic ARIA-H and therefore requires considerable caution, 5) particular vigilance is required to avoid administering thrombolytics to patients with ARIA-E presenting as a stroke mimic, which has resulted in several fatalities, and 6) in addition stopping antibody infusion, high-dose corticosteroids can be considered for treatment of severe ARIA. These vascular considerations will likely evolve with ongoing post-marketing data collection efforts.

